# Diseases of the Antrum of Highmore

**Published:** 1900-06-15

**Authors:** C. H. Oakman

**Affiliations:** Detroit, Mich.


DENTAL REGISTER.

Vol. LIV. JUNE 15, 1900. No. 6.

### REPORT OF THE PROCEEDINGS OF THE SOUTHWESTERN MICHIGAN DENTAL ASSOCIATION.

### DISEASES OF THE ANTRUM OF HIGHMORE.

BY C. H. OAKMAN, D.D.S., DETROIT, MICH.

(*Continued from May No., page* 264.)

*Case* 5.—The next was a male of fifty years of age, sent to me by his physician. The patient accidently discovered an opening leading to the antrum on the upper left side. While smoking he found he could force smoke into the antrum which would make its escape through the nose; he had had a tooth broken off during an attempted extraction, the roots of which were still present. I extracted them and enlarged the opening into the antrum. Shortly after the upper left second molar began to trouble him. As the second molar did not antagonize with the lower teeth it became elongated and loosened, so found it necessary to extract it, although the second molar was not responsible for any of the antral trouble.

Irrigated the antrum with warm sterile water and boracic acid solution, 2 percent cadmium sulphite, packing the antrum with gauze. Continued this treatment for two months. However, this was not a very severe case.

The patient concluded he was cured. I have not seen him after the two months' treatment. It was my method to keep a close watch of each case and never allow them to heal until every vestige of pus has been obliterated, seeing the patient occasionally from six weeks to two months after I think the case is cured.

In this instance I looked for a possible recurrence, as the patient was not sufficiently constant in his later treatment and ceased his visits, considering himself cured without my sanction.

The case in which the palatal bone was necrossed I frequently curetted, using cocain and chloride of ethel, stimulating the parts with aromatic sulphuric acid once a week.

On removing a portion of the palate the cleft immediately affected her speech, this was overcome by inserting a partial plate with two incisors; the teeth helping to keep the gauze in place. These antral cases should be treated at least once a day. Many require two or three treatments daily, much depending on quantity of pus I have found only two cases that were *other* than empyema. In the history of the cases only one could be traced to traumatic injury. Never found anything but staphylococcus present, so am rather inclined to believe that pus of this nature does not come in true catarrh.

A special syringe should be used in each case unless it be an all metal one as it is difficult to disinfect the inside of the rubber bulb without disintegrating the rubber.

I always have a Moffat syringe on hand for each case.

There is a very marked difference in the size of the antra In some the outer wall being depressed and difficult to drill without injuring the mucous membrane.

### DISCUSSION.

DR. RIX: My treatment of cases of this kind has been to first, by thorough curetting remove all necrotic tissue, and then by a thorough irrigation with aromatic sulphuric acid, remove all necrosed bone which may not have been reached by the knife. The acid serves not only to dissolve away the necrosed bone, but it also cauterizes and destroys dead soft tissues, and when diluted effectively stimulates the live tissues, hastening the nutritive processes and repair. This treatment I follow with simple antiseptic irrigation to keep the tissue free of irritating accumulations and let nature do the rest. As a systemic treatment, I prescribe Iodide of Potassium, with Stillingia and Sarsaparilla. The following formula may be used:

℞ Iodide of Potassium, - - - - ʒiij :
Tincture of Stillingia, - - - ℥ij :
Compound Syrup of Sarsaparilla, ℥jv :
M. Sig., a desertspoonful in water three times a day.

I never had a case so severe as some related by Dr. Oakman, I think I never treated one longer than eight weeks.

Dr. Watling: I do not understand why these cases were under treatment for so long a time. I never had a case that required over three months for treatment; except a case which was the result of, or at least aggravated by a specific systemic disorder. I do not believe in the extensive operations made by some surgeons, neither do I believe in much curetting; all I want is a free access and at suitable location to secure proper drainage. Extensive operations mean loss of tissue which it takes time to rebuild if it ever can be successfully accomplished. For the same reasons I would not advocate the use of solvent cauterizing drugs, sometimes a single application of sulphuric acid will be indicated; but much better results will generally be obtained from milder antiseptic and stimulating drugs, with an occasional application of a somewhat irritating astringent.

Great difficulty is often experienced in diagnosing these cases; if it were possible to make a satisfactory diagnosis of these conditions, it would simplify the treatment. As an illustration, related a case of a gentleman who came to the college clinic to have a treatment for antral trouble continued which had been operated for by a celebrated German surgeon, who left it with a silver drainage tube in place. After careful examination it was found that an absessed tooth was the cause of the antral discharge. The removal of the tooth very soon effected a cure, which had been delayed several years by an incomplete diagnosis.

Dr. Oakman: I should not use any irritating drugs in the treatment of antral troubles as they will surely keep up the suppuration, and there will be much loss of tissue. Such drugs as aromatic sulphuric acid and bichloride of mercury should not be used in any case; and if used, they should not be repeated at close intervals.

In the cases I have reported, there was no specific disease in any case, and I did all I possibly could to establish the fact in each case. In one case the patient was probably tubercular. The length of time consumed in the treatment of these cases seems great; but when the extent of the disease is taken into account it is not unusual, I am very sure neither of these cases could have been cured in six or eight weeks. And I am also confident that the treatment was materially shortened by the operation made, to say nothing of the relief to the patient which followed, and possibilities thus afforded for direct application of the treatment.

For lack of time the last paper on the program "The Teeth a Primary Cause in General Disease," by Dr. Essig, was not read.

Some interesting clinics were given; Dr. U. M. Richardson, packed the body and baked with the Peck

Electric Furnace, a piece of continuous gum, and, also made a porcelain faced crown for a central incisor, using platinum for the metal parts, soldering it with platinum solder and backing it with high fusing bodies; both were very skillfully done.

Dr. W. E. Harper, of Chicago, demonstrated his ideas of cavity preparation, and methods of packing gold fillings. The Doctor is a genius, and while he advocates some unusual methods, especially of cavity preparation, he is so enthusiastic about it, and gives such good reasons for his proceedures, that he tempts one to abandon his long cherished and tested methods for those which present enticing enducements to do so. This is one of the things which the man who will not attend the society meetings misses, as it can not be reported.

The doctor also showed a very nice method of making an all gold cap crown. It is intended to reproduce in gold a properly contoured crown modeled from a plastic material, with which a matrix is made which will reproduce by swaging the exact outline as represented in the model.

---

One of the happiest incidents of the meeting was the banquet and entertainment extended to the members of the Society by the citizens of Decatur. The banquet was held in one of the halls of the city, and was furnished by the ladies of the Universalist Church of the town. There were nearly forty at the banquet, and there was plenty of delicacies, such as only the ladies know how to provide. A number of toasts were made and responded to by the various guests.

Dr. N. E. Hooper, the host, responded in very cordially welcoming us to the hospitalities which the people of the town had provided for us.

Dr. Harper replied to Dr. Hooper's cordial welcome with a witty speech, in which he committed us to not only demonstrate the sincerity of our thanks for the generous hospitality shown us, by straining our capacities in order to do justice to the feast which was spread before us, but we were obligated to make ourselves perfectly at home, at any time of the day or night, while in the town.

Dr. Watling responded to the toast "The University." He gave a brief sketch of its career and of its prospective tendency, together with some facts as to its present condition; also spoke of its value to the State, the people, and especially to the dental profession, and urged that it be generously supported and utilized for the benefit of the people and the honor of the State.

Dr. Rix, in his inimitable manner, and with his ready Irish wit, spoke for "The Dentist at Home." He, very happily and feelingly, advocated the necessity of a dentist having a home. The value of a home, a wife, and children in keeping a man from brutalizing himself or going to the bad cannot be overstated. A man's home should be to him the dearest spot on earth. He should own his own home, he should prize it, not for its commercial value, but because it is the place where all that is dearest to him, his wife, spends most of her time, where the children are born, and reared and married, and to which the dear grandchildren love to come. "There is, indeed, no place like home, and the dentist's home should be the place of all others to him. I advise all you young men to get one. For forty years I have had the best one on earth and I know what I am talking about."

Dr. R. M. Speer, responded to the toast, "Our Wives and Sweethearts—may they never meet."

*Mr. Toastmaster, Ladies and Gentlemen:*—When I was asked to respond to this toast I supposed it referred to the better part of the married and single members of this association. The sentiment "may they never meet" was a poser, why should they never meet? Then I thought it must have emanated from the brain of some timid, would-be married man who was afraid of the pointers his sweetheart might receive. But now here comes that auburn sun-set of a toastmaster (Dr. Fowler was the toastmaster—Ed ) with the thought that "open confession is good for the soul," and tells you that he at least, once upon a time, at a summer resort had a combination of a wife and a sweetheart. It is no longer a poser, he might reverently add, please God may they never meet.

Mr. Toastmaster, possibly only a married man can, with partial success, discuss this subject, and even he can but approach it with fear and trembling, for if he should be caught between the two sometime inharmonious elements, or if the wife discovers him in the atmosphere of a sweetheart other than herself it would be unnecessary to state what would take place.

In that event the only thing he could do would be to borrow the remark made by the father-in-law of Mohamet (who was not sharp enough to outwit a woman) and console himself with the reflection that woman is a necessary evil and the greatest evil of them all is that she is necessary.

Then again Mr. Toastmaster I can see the fact that even a married man ought not to be called upon to respond to this sentiment, because if he draws upon his imagination as he will have to do to make a proper response many suspicious persons would most likely affirm that he is only relating his own personal experiences when in fact he does not have a small part of the information possessed by some bachelors who have sipped nectar from many flowers.

Our wives, God bless them, who in their simple trust believes that Stout telegraphed to meet him in Kalamazco, and he will pay the expense for you to buy two pennyweights of gold solder needed for the bridge next day. The reason you returned so late—a patient next day for a plate from the country; it had to be vulcanized; it was necessary to watch the vulcanizer as it leaked. Ah! if the hand that rocks the cradle controlled the night key to such pipe stories they would soon become obsolete.

But if a wife loses her place as sweetheart through her or his fault and another comes "two stepping" in, introduced by a cupid with cloven feet, then indeed may they never meet, for if they should how the hair would fly, the molars grind, the teeth clash and crash, the plates break, and crowns and fillings be scattered all over the floor.

If a wife should feel that her husband was inclined toward another sweetheart there are two ways of handling him; one is to make love to him as in days of old, another, stand him in the corner, maul him with a rolling-pin, a feather duster or some other dangerous weapon, I believe a dentist would prefer the first named remedy.

The truest, the dearest, the sweetest sweetheart of them all is the sweetheart of yesterday, the wife of to day, who remains the sweetheart all through the married life, in this event sympathy caresses the bowed head of sorrow and misfortune. The wrinkles on the face of old age are constantly smoothed by the white hands of love and the fire from the domestic hearth mingling with the warmth of loving hearts make home a place where blessings flow.

DR. J. C. ARNOLD responded to the toast "Gold Miners:"

*Mr. Toastmaster, Ladies and Gentlemen:*—When I learned that our toastmaster had assigned, and was depending upon me for a toast to-night, I wondered if he was somewhat of a phrenologist and had mistaken me for some other dentist who had a "bump" for giving toasts. He may have perhaps "told a fellow to get another fellow, and he got me." At any rate I'm here and I'd like to meet the fellow who is responsible for my being here, at the hotel immediately after the banquet is over.

An old proverb tells us "that a fool uttereth all his mind; but a wise man keepeth it till afterward." I trust that none of my friends will hesitate for one moment to at *least endeavor* to silence me if there is the least indication that I am uttering all my mind, for you all know it would be wiser for me to keep some of it "till afterwards." Now that the Spring elections are over and I not being successful in getting into office (not even to that of pathmaster), and not having the duties of a township or village officer to think over after office hours, I have silently resolved that after giving this toast on "Gold Miners," to be "one of them."

Firstly, because they are not apt to be called upon to give toasts upon subjects which they are unable to handle.

Secondly, when a gold miner digs his gold he has it, he is not like the dentist, who, if he is fortunate enough to dig five dollars ($5.00) in gold out of a whole year's practice, he must dig it a second time out of his pocket to pay his honest debts.

It is not the gold miner alone who digs for gold, we are all gold miners. It is true we do not all take our picks and go down into the mine in search of the precious metal, but yet we are mining for it each day.

We may compare the gold miner with any industrious individual in any walk in life, he does not always secure what he so much desires any more than the rest of us, and after his years of ceaseless toil he is repaid with a pile of the yellow metal; he cares not what "mortal man" does to effect a change in that metal so long as he realizes all that he has labored for; we care not whether a physical or a chemical change takes place in the gold, if we are fortunate enough to get some of the "change."

It is the same with the gold miner as it is with the rest of us, and his life is not without the discouragements and disappointments that come to us all.

It is lesson after lesson with the scholar, blow after blow with the laborer, crop after crop with the farmer, picture after picture with the artist and mile after mile with the traveler that secures the success that we all so much desire.

And surely, when success comes, it is indeed a mine of wealth that must be properly worked in order to be a paying investment, the same as any mine that is worked by the gold miner, whom we all wish success and long life, and may he always find all that glitters to be gold, and may he find sixteen (16) gold dollars to one (1) silver one.

As I said that, though none of us mine with the pick for gold, it is to be hoped that during our years of mining we will not forget the golden rule, that our barks will sail upon a golden sea and our sunsets be golden. And finally, when we are ready to cross to the golden shore, we will have no need for the following ad.: Lost—Somewhere between sunrise and sunset, two golden hours, each set with sixty diamond minutes—no reward is offered, as they are lost forever.

At the close of the banquet we adjourned to a large hall where a musical treat was given us by the Decatur Orchestra and a quartette. A large number of the citizens were present, and it did not take much encouragement by the waltzes and two-steps played by the orchestra to clear the floor and dancing was the general order, interspersed with singing of character songs by Drs. Fowler, Roe and other versatile members of the party. It was a fitting and highly enjoyable occasion.

Independent of the earnestness which characterizes the methods of conducting the discussion of subjects brought before this society, the social features are certainly to be commended.

The next meeting will be held the second Tuesday in

September, at St. Joseph, and by vote of the society the Northern Indiana Society has been invited to meet with the Southwestern.

